# Characteristics and determinants of sexual behavior among adolescents of migrant workers in Shangai (China)

**DOI:** 10.1186/1471-2458-9-195

**Published:** 2009-06-19

**Authors:** Shenghui Li, Hong Huang, Yong Cai, Gang Xu, Fengrong Huang, Xiaoming Shen

**Affiliations:** 1 Shangai Xin Hua Hospital affiliated with Shangai Jiaotong University School of Medicine, Shangai, PR China; 2 School of Public Health affiliated with Shangai Jiaotong University School of Medicine, Shangai, PR China; 3 Shangai Municipal Health Bureau, Shangai, PR China; 4 Key Laboratory of Children's Environmental Health, Shangai, PR China

## Abstract

**Background:**

China is facing a critical challenge of rapid and widespread human immunodeficiency virus (HIV)/acquired immunodeficiency syndrome (AIDS) increase. Rural-to-urban migration plays a crucial role in shifting the HIV/sexual transmitted infection (STI) epidemic. The purpose of this study was to assess the prevalence of sexual behaviors and the correlates among the early adolescents of migrant workers in China.

**Methods:**

A cross-sectional study was conducted in 10 junior high schools from April to June of 2008. A total of 2821 adolescents aged 14.06 ± 0.93 years (8.9% of migrant workers vs. 91.1% of general residents) participated in the survey. A self-administrated questionnaire was used to collect information on knowledge, attitude, and behaviors associated with increased risk for HIV/STI.

**Results:**

The percentage of adolescents who ever had sexual intercourse or had sexual intercourse in last three months was 7.2% and 4.3% in adolescents of migrant workers, respectively; in contrast, 4.5% and 1.8% in their peers of general residents, respectively. 47.3% adolescents of migrant workers and 34.3% of those adolescents of general residents reported no condom use in sexual intercourse during last three months. Multivariate logistic regression analyses found that migration was a independent risk factor for sexual intercourse in last three months in our sampled adolescents (odds ratio [OR] = 1.23, 95% confidence interval [CI]: 1.01–1.72). In adolescents of migrant workers, factors such as lower family income (OR: 2.22, CI: 1.09–3.05 for low level; OR:1.25, CI: 1.04–1.59 for medium level), younger age at first sexual intercourse (OR: 1.24, CI: 1.09–1.57), lower knowledge on HIV/AIDS (OR: 0.93, CI: 0.90–0.97), and fewer communication on HIV/AIDS related issues (OR: 0.79, CI: 0.90–0.97) were related to sexual intercourse in last three months.

**Conclusion:**

Based on these results, we advocated that heightened concerns targeting the adolescents of migrant workers be particularly necessary, given their higher level of sexual experience, lower socioeconomic status, restricted reproductive health information, and vulnerability to HIV/STI.

## Background

China is facing a critical challenge of rapid and widespread HIV/AIDS increase [1]. In 2007, the Chinese government along with the World Health Organization (WHO) and the Joint United Nations Programme on HIV/AIDS (UNAIDS) estimated that 700,000 people were living with HIV in China, including 85,000 AIDS patients [2]. The data from the national sentinel surveillance system shows that the HIV epidemic in China is still growing [3]. WHO warned that, if there were no effective preventive measures adopted, that the number of HIV/AIDS cases would reach 10 million in China by 2010 [3].

Adolescents and youths are at serious risk for HIV, accounting for half of new HIV infection in China each year [3]. Between 2001 and 2005, HIV/AIDS diagnoses increased more than 20% in people aged 13 to 24 years [4], and the proportion of those diagnosed with AIDS continues to increase [5]. In addition, adolescents and youths aged 15 to 24 years represent approximately 25% of sexually active persons and accounted for nearly 50% of new STI cases [6]. Therefore, adolescents and youths are important subpopulation for sexually-related prevention and intervention efforts.

Data also demonstrated that the so-called 'floating population' – the approximately 150 million migrant workers and 73% of them come from poorer regions of the country and work in the cities as laborers, restaurant workers and sex workers has acted as the 'tipping point' for the HIV/STI epidemic in China [7,8]. It was shown that rural-to-urban migration may play a crucial role in shifting the HIV/STI epidemic by broadening social and sexual mixing [9,10]. In addition, migrants' perceived HIV risk, knowledge of HIV, and rates of condom use were low [11]. Therefore, compared to general residents in urban areas, HIV/STI prevalence among rural-to-urban migrants was relatively high [12]. With the ongoing economic development and industrialization in China, more and more rural residents have migrated to urban area for better job and better living environment.

In the past 15 years, the massive migration, mostly from rural to urban areas, especially large metropolises, has taken place in China, which constitutes what has been labelled the largest labour flow in human history [13]. According to official censuses, the migrant population in China increased from 6.57 million in 1982 to 48.41 million in 1995 and 144.39 million in 2000 [14,15]. In 2005, a sample survey showed that the migrant population had reached 147.35 million (National Bureau of Statistics of China, 2006). Shangai, as one of the biggest economic centres in China, shared approximately above 15% of the total national migrant population in 2005 (National Bureau of Statistics of China, 2006).

Under Chinese insurance system, migrants are mostly excluded from the urban employee's basic insurance scheme. Therefore, migrant workers face barriers to accessing education and healthcare, which was a major public concern in China. The same issue also existed in their second generation. For a long time, children of migrant workers were excluded from urban public school. Moreover, they also exposed other risk factors, such as lower socioeconomic status, special family environments, and frequent migration.

It was well known that different sociocultural groups (different age groups, different educational levels, different cultural background, from rural to urban areas, migrants and permanent residents) varied relative to their sexual attitude and behaviors [16-20]. Adolescents of migrant workers, as a special group in urban, may have greater exposure to risk factors than their peers of general residents, including lower socioeconomic status, special family environments, limited access to regular and formal education because of accommodation varies, and limited access to health care. Despite its importance, however, to the best of our knowledge, there was no comprehensive research on HIV/STI-related risk behaviors in the group up to now.

To our knowledge, this is the first study of HIV/STI-related risk behaviors in adolescents of migrant workers in China. In this article, we mainly address two questions:

◆ Prevalence: among adolescents in the sample, what proportion reported each HIV/STI-related risk behavior? Compared to adolescents of general residents, were HIV/STI-related risk behaviors in adolescents of migrant workers more prevalent?

◆ Correlating factors: what factors can predict sex behaviors? Were there differences in correlating factors of sex behaviors between adolescents of migrant workers and their peers of general residents?

## Methods

### Sample and Procedure

There are twenty districts in Shangai and migrant workers mainly live in six districts. From the six districts, two districts were selected for the study sample on the basis of cluster-stratified selection procedure, using geographic location, economic standard, and population density as criteria. For every district, 5 junior high schools with higher proportion of children of migrant workers were selected. All the students in selected schools yielded the study sample. Of 3104 students eligible for the study, 2821 (90.9%) returned completed questionnaires. The mean age of the final sample was 14.06 years (SD: 0.93; range:11.08–16.67 years); 48.5% were males and 51.5% were females.

This study was conducted from April to June of 2008. The research aims were explained to school principals and teachers of the target schools. Permission was obtained to carry out the study, which is the usual practice in China. An anonymous questionnaire was given to the students during counseling hours. Researchers explained the study purpose to the students and emphasized that participation was voluntary.

The study was approved by the Ministry of Education of the People's Republic of China.

### Measure

#### HIV/STI-related risk behaviors

The Adolescents' Reproductive Health Questionnaire (ARBQ) was used to collect information on adolescents' knowledge, attitude, and behaviors concerning HIV/STI. The ARBQ was a self-administrated questionnaire, which was developed based on literature review [9,10,12,19,21-27], qualitative research, pilot studies, and reliability assessment. The final version of the questionnaire was conceptually grouped into 6 subscales: (1) knowledge on sex/HIV/AIDS (31 items); (2) attitude toward sexual/drug-use behaviors (8 items); (3) attitude toward person with HIV/AIDS (7 items); (4) exposure to sexual/drug-use behaviors (11 items); (5) self-protection efficacy (4 items); and (6) communication about sex/HIV/AIDS issues (4 items). The internal consistency of overall questionnaire and the six subscales was good (Cronbach's alpha coefficient was 0.76 for overall questionnaire and ranged from 0.48 to 0.87 for six subscales).

##### (1) Subscale of knowledge on sex/HIV/AIDS

To assess participant's knowledge level on information about HIV, ways by which HIV is transmitted, ways by which HIV is not transmitted, self-protection information, and progress in HIV prevention/cure/epidemic, 31 sex/HIV/AIDS related items were schemed in the questionnaire. For example, do you consider the more sexual partners, the higher risk of HIV infection? Do you consider using condoms in sexual intercourse can reduce the risk of HIV infection? and etc. Answer ranged by a 3-point scale: "yes", "do not know", and "no". Correct answers were credited with a score of one and incorrect answer or responding "do not know" with a score of zero. The subscale score was then calculated as the higher the score, the higher level the knowledge. The internal consistency of the subscale was Cronbach's alpha coefficient = 0.87.

##### (2) Subscale of attitude toward sexual/drug-use behaviors

Eight items were used to evaluate participant's attitude toward sexual/drug-use behaviors, including premarital sex, multiple-partner sex, injection drug use, alcohol drinking, and etc. Answers ranged by a 3-point scale: "disagree", "somewhat disagree", and "agree". For each statement, "disagree" was scored with two point, "somewhat disagree" was scored with one point, and "agree" was scored with zero. The subscale scores were then calculated as the higher the score, the more positive and conservative the attitude. The internal consistency of the subscale was Cronbach's alpha coefficient = 0.86.

##### (3) Subscale of attitude toward person with HIV/AIDS

This subscale was programmed to evaluate participant's attitude toward person with HIV/AIDS. Items, such as whether or not willing to study/eat together with person with HIV/AIDS, whether or not willing to keep secret for person with HIV/AIDS, and etc, were included in the subscale. Answers ranged by a 3-point scale: "agree", "somewhat agree", and "disagree". For each statement, "agree" was scored with two point, "somewhat agree" was scored with one point, and "disagree" was scored with zero. The subscale score was then calculated as the higher the score, the more friendly the attitude. The internal consistency of the subscale was Cronbach's alpha coefficient = 0.48. 0.48 is not high enough for the internal consistency. However, based on the following two reasons, we thought to keep the subscale of attitude toward person with HIV/AIDS in the final ARBQ: i The items of the subscale could provide information on Chinese adolescents' attitude toward person with HIV/AIDS; ii From the point of statistics, 0.7 and above was acceptable for the internal consistency of a scale. However, clinical significance must be taken into account when we select or omit a item. To the best of my knowledge, some scales or questionnaires have subscales with internal consistency under 0.7, even under 0.5 [28].

##### (4) Subscale of exposure to sexual/drug-use behaviors

There were 9 items respect to sexual and drug use behaviors in the questionnaire. In addition, we also examined alcohol and cigarette use because they can lower inhibitions and increase the likelihood of sexual and drug use behaviors (2 items). The internal consistency of the subscale was Cronbach's alpha coefficient = 0.73.

##### (5) Subscale of self-protection efficacy

Four items were used to evaluate participant's self-protection efficacy. For example, if unwilling to have sex, are you able to refuse? Are you able to adopt measures to protect yourself from pregnancy and infection during sex intercourse? and etc. Answers ranged by a 3-point scale: "yes", "do not know", and "no". For each statement, "yes" was scored with two point, "do not know" was scored with one point, and "no" was scored with zero. The subscale score was then calculated as the higher the score, the higher level of protection self-efficacy. The internal consistency of the subscale was Cronbach's alpha coefficient = 0.59.

##### (6) Subscale of communication about sex/HIV/AIDS issues

Communication about sex/HIV/AIDS issues was measured by inquiring how often the participants discussed sex/HIV/AIDS issues with parents, peers, teachers, and doctors/health experts? Answers were on a 3-point scale, with 3 being "usually", 2 being "sometimes", and 1 being "rarely/no" The subscale score was then calculated as the higher the score, the higher level of communication. The internal consistency of the subscale was Cronbach's alpha coefficient = 0.78.

#### Sociodemographic characteristics

This section consists of participant's gender, age, grade, parents' education levels, household income [RMB(yuan)/person/month], parents' relationship, and family structure (i.e., single parent family, nuclear family, or large family). The score of each sociodemographic characteristic are demonstrated in Table 1.

**Table 1:** The score of each sociodemographic characteristic of the sampled participants

Sociodemographic variables	Score			
	
	1	2	3	4
	
Gender	Male	Female		
Grade	One	Two	Three	
Family income ^#^	Low (<1000)	Medium (1000–2499)	High (≥ 2500)	
Family structure	Single parent family	Nuclear family	Large family	
Parents' relationship	Bad	Not bad	Good	
Father's education level	Illiterate/primary	Middle school	High school	College and above
Mother's education level	Illiterate/primary	Middle school	High school	College and above

^#^Family income was expressed in RMB(yuan)/person/month

In our study, a family including children, parents, grandfather and/or grandmother, who live together, was defined as a large family; a family including children and parents was defined as nuclear family; and a family only including children and father or mother was defined as a single-parent family.

In China, there are no established systems to assess the socioeconomic status (SES) of individual family. Therefore, parental education levels and household income were used as indicators of the family SES.

### Statistical Analysis

Statistical descriptions were made by use of the mean, standard deviation and percentages. Demographic differences between study groups were analyzed by Independent-samples t test and the Chi-square test. Independent-samples t test and the Chi-square test were also used to compare score differences and prevalence of sexual/drug-use behaviors between study groups, respectively.

Multivariate logistic regression analyses were performed to examine the correlating factors of sexual intercourse within last three months, with the dependent variable designated as "1" if there was sexual intercourse within last three months and "0" if there was no sexual intercourse within last three months. The final multivariate model included variables retaining significance after a forward likelihood-ratio stepwise elimination procedure. Statistical tests of regression estimates or OR were based on Wald statistics.

All analyses were performed using the Statistical Package for Social Sciences (SPSS) for Windows, version 12.5. In the calculation and presentation of the results, the statistical significance was set at p < 0.05 (two tailed).

## Results

### Demographic characteristics of the study sample

In China, under the current *hukou *system (household registration system), every person has a permanent place of household registration (either an agriculture status or a nonagricultural status) that can only be changed with official approval [29]. The migrant workers refer to those people who left their places of household registration – poor rural regions of the country and work in the cities as laborers, restaurant workers, factory workers, and etc. Our survey showed that migrant workers worked in Shangai mainly came from rural regions of, in rank order, Jiangsu Province, Henan Province, Anhui Province, Zhejiang Province, and Sichuan Province. In the present study, immigrants coming from other urban areas were not included in the group of migrant workers.

Of our sample, adolescents of migrant workers referred to those adolescents who came from the rural region and his/her mother or father served as migrant workers in the city. Our study revealed that 8.9% of the sample (n = 252) belonged to the defined subgroup. Of all these adolescents, 10.4% stayed in Shangai for less than one year, 14.1% stayed for 1–2 years, 75.5% stayed for 3 or above years.

Table 2 summarized the demographic characteristics of the study sample. No significant differences were observed in gender and family structure between the two groups. However, there was statistically significant difference in age, family income, father's educational level, and mother's educational level between the two groups. There was the tendency of older age (t = 3.28, p < 0.01), lower family income (*χ*^2 ^= 181.59, p < 0.01), lower father's educational levels (*χ*^2 ^= 305.89, p < 0.01), and lower mother's educational levels (*χ*^2 ^= 420.16, p < 0.01) in adolescents of migrant workers.

**Table 2:** Demographic characteristics of study participants by study groups

	Adolescents of migrant workers (N = 252)	Adolescents of general residents (N = 2569)	*t*/*χ*^2^
Age (years, mean ± SD)	14.17 (1.03)	13.99 (0.92)	3.28** ^a^
Gender (%)			1.97^b^
Males	50.3	48.0	
Females	45.8	52.0	
Family income (%) ^#^			181.59** ^c^
Low (<1000)	45.5	20.1	
Medium (1000–2499)	50.1	34.0	
High (≥ 2500)	14.5	45.9	
Family structure			3.23* ^c^
Single parent family	18.5	9.3	
Nuclear family	45.5	50.0	
Large family	35.9	40.7	
Father's education level (%)			305.89** ^c^
Illiterate/primary	8.9	1.2	
Middle school	45.0	15.4	
High school	38.3	42.8	
College and above	7.8	40.6	
Mother's education level (%)			420.16** ^c^
Illiterate/primary	20.2	2.5	
Middle school	48.6	20.1	
High school	26.2	41.6	
College and above	5.0	35.7	

^#^Family income was expressed in RMB(yuan)/person/month

^a ^Independent-samples t test

^b ^2*2 Chi-square Test

^c ^K*2 Chi-square Test

* *P *< 0.05

** *P *< 0.01

### Prevalence of sexual/drug-use behaviors in the study sample

Table 3 compared the differences in the prevalence of sexual behaviors and substance use between the two groups. There were statistically significant differences between the two groups of adolescents in the lifetime sexual intercourse, age of the first sexual intercourse, sexual intercourse within last three months, unprotected sexual intercourse, and cigarette smoking. Meanwhile, other sexual/substance use behaviors, such as sex while drunk, sex with high-risk partner, drug use, and alcohol drinking were not found to be statistically different between the two groups.

**Table 3:** Prevalence of HIV/STI-related risk behaviors: differences by study groups

	Adolescents of migrant workers (N = 252)	Adolescents of general residents (N = 2569)	*χ*^2^
I Sexual intercourse in lifetime (%)			3.52* ^a^
Ever had	7.2	4.5	
Never had	92.8	95.5	
II Age of the first sexual intercourse (%)			4.95* ^b^
11–12 years	11.1	5.8	
13–14 years	60.0	29.7	
≥ 15 years	28.9	64.5	
III Sexual intercourse in past 3 months (%)			3.45* ^a^
Ever had	4.3	1.8	
Never had	95.7	98.2	
IV Among sexual intercourses within past 3 months			
1. Partners >1 (%)			0.69^a^
Ever had	7.1	6.9	
Never had	92.9	93.1	
2 Unprotected sexual intercourse (%)			6.31** ^a^
Ever had	47.3	34.3	
Never had	52.7	65.7	
3. Sexual intercourse while drunk (%)			0.79^a^
Ever had	7.1	6.7	
Never had	92.9	93.3	
4. Sexual intercourse with high-risk partners (%)			0.03^a^
Ever had	9.1	7.6	
Never had	90.9	92.4	
V Substance use			
1. Injection drug use (%)			0.21^a^
Ever had	0.7	0.4	
Never had	99.3	99.6	
2. Oral/rhinal drug use (%)			0.31^a^
Ever had	0.7	0.4	
Never had	99.3	99.6	
3. Cigarette smoking (%)			7.06** ^a^
No/occasional	97.9	99.6	
Often/usually	3.1	0.8	
4. Alcohol drinking (%)			0.12^a^
No/occasional	99.3	99.0	
Often/usually	0.7	1.0	

Unprotected sexual intercourse refers to the sexual intercourse without condemn use

High-risk partners refer to people with HIV/AIDS/STI, people with drug use, and people with

multiple sexual partners

^a ^2*2 Chi-square Test

^b ^K*2 Chi-square Test

* *P *< 0.05 ** *P *< 0.01.

In contrast, sexual intercourse in lifetime (7.2% vs. 4.5%, *χ*^2 ^= 3.52, p < 0.05), sexual intercourse within last three months (4.3% vs. 1.8%, *χ*^2 ^= 3.45, p < 0.05), unprotected sexual intercourse within last three months (47.3% vs. 34.3%, *χ*^2 ^= 6.31, p < 0.01) and frequent cigarette smoking (3.1% vs. 0.8%, *χ*^2 ^= 7.06, p < 0.01) were more prevalent in adolescents of migrant workers. In addition, compared to their peers of general residents, adolescents of migrant workers tended to have sexual intercourse in younger age (*χ*^2 ^= 4.95, p < 0.05).

### Scores in knowledge, attitude, protection self-efficacy, and communication in the study sample

Table 4 showed the scores in knowledge, attitude, protection self-efficacy, and communication in adolescents by study groups. It shows that there were no statistical differences existed in attitude, protection self-efficacy, and communication between the two groups. However, scores in knowledge were statistically significantly lower in adolescents of migrant workers than their peers of general residents (15.40 vs, 18.89, t = 2.95, p < 0.05).

**Table 4:** Scores in knowledge, attitude, self-efficacy, and communication: differences by study groups (mean ± SD)

	Adolescents of migrant workers (N = 252)	Adolescents of general residents (N = 2569)	*t*	*P-value*
Scores in HIV/AIDS knowledge (% in total score)				
Total	15.40 ± 6.10 (46.77)	18.89 ± 6.20 (60.93)	2.95^a^	0.02
Male	16.12 ± 6.50 (52.00)	18.77 ± 6.47 (60.55)		
Female	14.34 ± 5.46 (46.25)	19.20 ± 5.67 (61.94)		
*t*	0.77^a^	-1.77^a^		
*P-value*	0.46	0.08		
Scores in attitude toward sex behaviors (% in total score)				
Total	14.52 ± 2.62) (90.75)	14.27 ± 2.86 (89.19)	-1.06^a^	0.29
Male	14.01 ± 3.21) (87.56)	13.82 ± 3.22 (86.38)		
Female	15.05 ± 1.72) (94.06)	14.73 ± 2.35 (92.06)		
*t*	-2.32^a^	-8.15^a^		
*P-value*	0.02	0.00		
Scores in attitude toward person with HIV/AIDS (% in total score)				
Total	10.07 ± 2.73 (71.93)	10.01 ± 2.82 (71.50)	-0.25^a^	0.80
Male	10.20 ± 2.55 (72.86)	10.02 ± 2.91 (71.57)		
Female	9.98 ± 3.01 (71.29)	10.04 ± 2.75 (71.71)		
*t*	0.46^a^	-0.20^a^		
*P-value*	0.64	0.84		
Scores in protection self-efficacy (% in total score)				
Total	5.41 ± 1.54 (67.62)	5.30 ± 1.39 (66.25)	0.86^a^	0.39
Male	5.26 ± 1.74 (65.75)	4.99 ± 1.63 (62.38)		
Female	5.60 ± 1.31 (70.00)	5.62 ± 1.04 (70.25)		
*t*	-1.25^a^	-11.14^a^		
*P-value*	0.21	0.00		
Scores in communication on sex/HIV/AIDS issue (% in total score)				
Total	0.89 ± 1.47 (11.13)	1.08 ± 1.50 (13.50)	0.87^a^	0.38
Male	0.91 ± 1.54 (11.38)	1.04 ± 1.54 (13.00)		
Female	1.03 ± 1.39 (12.88)	1.10 ± 1.46 (13.75)		
*t*	-1.88^a^	-0.21^a^		
*P-value*	0.83	0.06		

Scores in HIV/AIDS knowledge, attitude toward sex behaviors, attitude toward person with HIV/AIDS, protection self-efficacy, and communication on sex/HIV/AIDS issue were normally distributed with statistics of Shapiro-Wilk = 0.93 (*P *< 0.01), 0.78 (*P *< 0.01), 0.95 (*P *< 0.01), 0.79 (*P *< 0.01), and 0.83 (*P *< 0.01), respectively.

^a ^Independent-samples t test

Among all adolescents, compared to males, females tended to have higher scores in attitude toward sexual behaviors (15.05 vs, 14.01, t = -2.32, p < 0.05 for adolescents of migrant workers; 14.73 vs, 13.82, t = -8.15, p < 0.01 for adolescents of general residents) and among adolescents of general residents, females tended to have higher scores in protection self-efficacy (5.62 vs, 4.99, t = -11.14, p < 0.01).

### Correlating factors of sexual intercourse by study groups

Multivariate logistic regression analyses were performed to examine the correlating factors of sexual intercourse in last three months in adolescents by study groups (see Additional file 1).

After controlling for some cofounders, multivariate logistic regression analyses found that migration was a risk factor for sexual intercourse in last three months in our sampled adolescents (OR = 1.23, p < 0.05). Five factors were significantly associated with an increased likelihood of sexual intercourse in last three months in adolescents of migrant workers: older age (OR = 1.51, p < 0.05), lower family income (OR = 2.22, p < 0.01 for low level; OR = 1.25, p < 0.05 for medium level), younger age of the first sexual intercourse (OR = 1.24, p < 0.05), lower scores in knowledge (OR = 0.93, p < 0.05), and lower scores in communication (OR = 0.79, p < 0.05). For adolescents of general residents, five factors were significantly associated with an increased likelihood of sexual intercourse in last three months: older age (OR = 2.04, p < 0.01), higher frequency of antecedent sexual behaviors (OR = 3.21, p < 0.01), higher frequence of alcohol drinking (OR = 2.23, p < 0.01), lower scores in attitude toward sexual/drug-use behaviors (OR = 0.88, p < 0.01), and lower scores in protection self-efficacy (OR = 0.75, p < 0.01).

## Discussion

The present study found that, compared to their peers of general residents, adolescents of migrant workers were more prevalent in sexual intercourse during lifetime (7.2% vs, 4.5%) and sexual intercourse during the last three months (4.3% vs, 1.8%). Among the adolescents with a history of sexual intercourse in last three months, more adolescents of migrant workers reported unprotected intercourse (47.3% vs, 34.3%). Moreover, our study demonstrated that, compared to their peers of general residents, adolescents of migrant workers tended to have sexual intercourse in younger age and more frequent cigarette smoking (as shown in Table 3). There results indicated that, compared to their peers of general residents, adolescents of migrant workers were at greater risk for HIV/STI.

A number of studies in China revealed that the prevalence of lifetime sexual intercourse ranged from 1.3% to 4.8% in general junior school students [21-23], which was consistent with the results of our study in adolescents of general residents. In studies of other countries, adolescents who ever had sexual intercourse were 48.7% in America (aged 10–24 years) [30], 38% in Italy (aged 14–19 years) [31], 17–46% in South Africa (aged 13–17 years) [32], 11% in Burkina Faso (aged 12–19 years) [33], 18–22% in Nigeria (aged 15–19 years) [34], and 5.1–56.6% in Turkey (aged 16–20 years) [35], which was higher prevalent than our sample (aged 11.08–16.67 years). The disparity may be due to different sample characteristics, different traditional cultural background, and different socioeconomic environment. For example, compared to the Western countries, Chinese society has a strong tradition of Confucianism, in which conservative values are prevailed. In such a traditional culture, abstinence is emphasized for unmarried people, especially for females, which is considered to be linked to personal and family honor. The intrinsic sociocultural values may partly explain the lower prevalence of sexual intercourse in Chinese adolescents.

Previous studies revealed that many factors were associated with adolescents' sexual intercourse: age, ethnicity, gender, substance use, family structure and socioeconomic status [13-17]. For example, a recent study in Ethiopia youth indicated that lower socioeconomic status and restricted educational were risk factors for high-risk sexual behaviors [17]. Adolescents of migrant workers mostly lived in an unstable and poor economic environment, which prevent them from getting regular and high quality education and health care. Our survey showed that, compare to their peers of general residents, adolescents of migrant workers were poor in knowledge on sex/HIV/STI and lower in awareness to risk behaviors (Table 4). In additional, our survey found that 18.7% of families was single parented in adolescents of migrant workers, which may lead to less parental caring, love, and monitoring for them and then resulted in more risk behaviors. The clustering of risk factors may partly or totally account for the differences in sexual behaviors and substance use between adolescents of migrant workers and their peers in general residents. It should be specially emphasized that unprotected sex in our sampled adolescents, especially in adolescents of migrant workers, was more prevalent than their peers of other countries [30,35-37], which indicated a higher level of vulnerability to a range of undesirable consequences associated with sexual activity, including HIV, STI, even unintended pregnancy.

To best address sexually related public health concern for adolescents, it is crucial to reveal the correlating factors, especially for higher-risk groups, in China. Consistent with previous studies, **o**ur study found that sexual behaviors were a multifactorial issue. In the present study, it was demonstrated that five variables were significantly associated with sexual intercourse in last three months for adolescents of migrant workers: older age, lower family income, younger age at first sexual intercourse, lower knowledge on HIV/AIDS, and fewer communication.

Among all the factors identified associated with sexual intercourse in last three months for adolescents of migrant workers, poor family income had the highest OR. Our survey showed that nearly one-half of families lived in extremely poor condition, with an average family income < 1000 RMB (yuan)/person/month. Such lower economic status could undoubtedly prevent children from having basic health care and educational improvement. Under the current educational system in China, education in primary school and junior high school was free-feed and compulsory, however, for a long time, children of migrant workers were excluded from the compulsory educational scheme in urban area. Although the problem was gradually resolved from last year in Shangai, children of migrant workers still face barriers to accessing regular education because of parents' frequent migrant. Therefore, although most schools provide HIV/STI education, children of migrant workers are unlikely to receive school-based intervention. That may be why our study found that lower knowledge on HIV/AIDS and fewer communication were strong factors associated with sexual intercourse in last three months for adolescents of migrant workers.

There are several limitations that should be considered in interpreting these results. Firstly, social-desirability bias and inaccuracy may be existed in answering the questionnaires despite guaranteed anonymity. Secondly, because the sample was not large enough, the analyses were not conducted by dividing the sample by age. As the age-range is quite spread, it would be possible that different factors are associated to risk sexual intercourse according to the age. Thirdly, China is a multi-ethnical country (56 ethnic groups) with a population of 1.3 billion (90.56% Han ethnicity vs, 9.44% other ethnicity named ethnic minority, such as Muslim ethnicity, Mongolia ethnicity, Uighur ethnicity, and etc). It is possible that, although may be very small, migrant workers were likely to be from ethnic minority. It was a pity that our study did not investigate the information on ethnicity of sampled participants. The forth limitation lies in the fact that our findings regarding migrant adolescents were much more so within the local-national environment and may not reflect the practice in other countries. In addition, the sample of adolescents of migrant workers was small, which may weaken the strengths of the study. The fifth limitation existed in study design. The cross-sectional nature of the study made it difficult to determine a causal relationship. Finally, our sample limited our analyses of demographic subgroups and can not be generalized to other population.

## Conclusion

It was a strength of the present study that we firstly focused on adolescents of migrant workers, an understudied subgroup with potentially greater risk for HIV/STI in China. Our study not only examined the characteristics of sexual behaviors in the subgroup but also explored the correlating factors. The results can provide data to promote specific intervention for improving their behavioral and sexual health. In addition, the comparative study between adolescents of migrant worker and their peers of general residents entailed an unusually rich data-set for interpreting the topic of adolescents' sexual behaviours in China.

## Abbreviations

HIV: human immunodeficiency virus; STI: sexual transmitted infection; AIDS: acquired immunodeficiency syndrome; WHO: the World Health Organization; UNAIDS: the Joint United Nations Programme on HIV/AIDS; ARBQ: the Adolescents' Reproductive Health Questionnaire; SES: socioeconomic status; SPSS: the Statistical Program for Social Sciences; OR: odds ratio; CI: confidence interval; NS: no significance.

## Conflicts of interests

The authors declare that they have no competing interests.

## Authors' contributions

All authors contributed the design of this research. SL drafted the manuscript and has been involved in the interpretation of the data. HH performed statistical analyses. GX, YC and FH played a major role in the field survey. XS made a substantial contribution to the interpretation of the data and has been involved in revising manuscript. All authors read and approved the final manuscript.

## Pre-publication history

The pre-publication history for this paper can be accessed here:



## Supplementary Material

Additional file 1 **Factors predicting sexual intercourse during past 3 months by logistic regression models: differences by study groups**. The data provided the results of Multivariate logistic regression analyses regarding correlating factors of sexual intercourse within last three months in adolescents of migrant workers and their peers of general residents. Click here for file
