# Supplementary material for: Characteristics and determinants of sexual behavior among adolescents of migrant workers in Shangai (China)
Source: BMC Public Health. 2009 Jun 19;9:195. doi: 10.1186/1471-2458-9-195 (PMC2706248; doi:10.1186/1471-2458-9-195)
Supplement: Additional file 1 — Factors predicting sexual intercourse during past 3 months by logistic regression models: differences by study groups. The data provided the results of Multivariate logistic regression analyses regarding correlating factors of sexual intercourse within last three months in adolescents of migrant workers and their peers of general residents. [file 1471-2458-9-195-S1.doc]

**Table 5.** Factors predicting sexual intercourse during past 3 months by logistic regression models: differences by study groups

|  | Adolescents of  migrant workers  (N=252) | |  | Adolescents of  general residents  (N=2569) | |  | The total adolescents  (N=2821) | |
| --- | --- | --- | --- | --- | --- | --- | --- | --- |
|  | B | OR(95%CI) |  | B | OR(95%CI) |  | B | OR(95%CI) |
| Migration |  |  |  |  |  |  |  |  |
| Yes |  | - |  |  | - |  | 0.21 | 1.23 (1.01-1.72) * |
| No |  | - |  |  | - |  |  | 1.00 |
| Age | 0.41 | 1.51 (1.01-2.26) * |  | 0.71 | 2.04 (1.71-2.44) ** |  | 0.68 | 1.88 (1.51-2.34) ** |
| Gender |  |  |  |  |  |  |  |  |
| male |  | NS |  |  | NS |  |  | NS |
| Female |  | 1.00 |  |  | 1.00 |  |  | 1.00 |
| Father’s educational |  |  |  |  |  |  |  |  |
| Middle school and below |  | NS |  |  | NS |  |  | NS |
| High school |  | NS |  |  | NS |  |  | NS |
| College and above |  | 1.00 |  |  | 1.00 |  |  | 1.00 |
| Mother’s educational |  |  |  |  |  |  |  |  |
| Middle school and below |  | NS |  |  | NS |  |  | NS |
| High school |  | NS |  |  | NS |  |  | NS |
| College and above |  | 1.00 |  |  | 1.00 |  |  | 1.00 |
| Family income |  |  |  |  |  |  |  |  |
| Low (<1000) | 0.41 | 2.22 (1.09-3.05) ** |  |  | NS |  | 0.43 | 2.89 (1.42-4.01) ** |
| Medium (1000-2499) | 0.18 | 1.25 (1.04-1.59) * |  |  | NS |  |  | NS |
| High (≥2500) |  | 1.00 |  |  | 1.00 |  |  | 1.00 |
| Antecedent sexual behaviors |  |  |  |  |  |  |  |  |
| Often/usually |  | NS |  | 1.01 | 3.21 (1.67-5.01) ** |  | 0.56 | 2.28 (1.22-4.83) ** |
| No/occasional |  | NS |  |  | 1.00 |  |  | 1.00 |
| Cigarette smoking |  |  |  |  |  |  |  |  |
| Often/usually |  | NS |  |  | NS |  |  | NS |
| No/occasional |  | 1.00 |  |  | 1.00 |  |  | 1.00 |
| Drunk |  |  |  |  |  |  |  |  |
| Often/usually |  | NS |  | 0.56 | 2.23 (1.31-3.89) ** |  |  | NS |
| No/occasional |  | 1.00 |  |  | 1.00 |  |  | 1.00 |
| Drug use |  |  |  |  |  |  |  |  |
| Yes |  | NS |  |  | NS |  |  | NS |
| No |  | 1.00 |  |  | 1.00 |  |  | 1.00 |
| Close relationship with high- risk persons |  |  |  |  |  |  |  |  |
| Yes |  | NS |  |  | NS |  |  | NS |
| No |  | 1.00 |  |  | 1.00 |  |  | 1.00 |
| Age of first sexual intercourse |  |  |  |  |  |  |  |  |
| <15 | 0.30 | 1.24 (1.09-1.57) * |  |  | NS |  | 0.42 | 1.36 (1.02-1.88) * |
| ≥15 |  | 1.00 |  |  | 1.00 |  |  | 1.00 |
| Score 1 | -0.07 | 0.93 (0.90-0.97) * |  |  |  |  |  | NS |
| Score 2 |  | NS |  | -0.13 | 0.88 (0.82-0.94) ** |  |  | NS |
| Score 3 |  | NS |  |  |  |  |  | NS |
| Score 4 |  | NS |  | -0.28 | 0.75 (0.65-0.83) ** |  |  | NS |
| Score 5 | -0.11 | 0.79 (0.66-0.92) * |  |  |  |  |  | NS |

Family income was expressed in RMB(yuan)/person/month.

Antecedent sexual behaviors refer to petting, kiss, and masturbation.

Score 1: scores in subscale of HIV/AIDS knowledge

Score 2: scores in subscale of attitude toward sex behaviors

Score 3: scores in subscale of attitude toward person with HIV/AIDS

Score 4: scores in subscale of protection self-efficacy

Score 5: scores in subscale of communication on sex/ HIV/AIDS issue

OR, odds ratio

CI, confidence interval

NS: no significance

* *P*<0.05

** *P*<0.01
